# The association between allostatic load and lymphedema in breast cancer survivors

**DOI:** 10.1007/s00520-025-09362-4

**Published:** 2025-03-21

**Authors:** Barnabas Obeng-Gyasi, Yevgeniya Gokun, Mohamed I. Elsaid, JC Chen, Barbara L. Andersen, William E. Carson, Sachin Jhawar, Jesus D. Anampa, Dionisia Quiroga, Roman Skoracki, Samilia Obeng-Gyasi

**Affiliations:** 1 Indiana University School of Medicine, Indianapolis, IN USA; 2 Center for Biostatistics, College of Medicine, The Ohio State University, Columbus, OH USA; 3 Department of Biomedical Informatics, College of Medicine, The Ohio State University, Columbus, OH USA; 4 Division of Medical Oncology, Department of Internal Medicine, The Ohio State University, Columbus, OH USA; 5 Division of Surgical Oncology, Department of Surgery, The Ohio State University, Columbus, OH USA; 6 Department of Psychology, The Ohio State University, Columbus, OH USA; 7 Department of Radiation Oncology, The Ohio State University, Columbus, OH USA; 8 Department of Medical Oncology, Montefiore Medical Center, Albert Einstein College of Medicine, Bronx, NY USA; 9 Division of Reconstructive Oncologic Plastic Surgery, Department of Plastic Surgery, The Ohio State University, Columbus, OH USA

**Keywords:** Breast cancer, Allostatic load, Lymphedema, Stress, Survivorship

## Abstract

**Purpose:**

Allostatic load, a measure of physiological dysregulation secondary to chronic exposure to socioenvironmental stressors, is associated with 30-day postoperative complications and mortality in patients with breast cancer. This study aimed to examine the association between allostatic load (AL) at diagnosis and development of breast cancer-related lymphedema (BCRL).

**Methods:**

Patients aged 18 years or older who received surgical treatment for stage I-III breast cancer between 2012 and 2020 were identified from The Ohio State University Cancer Registry. AL was calculated using biomarkers from the cardiovascular, metabolic, renal, and immunologic systems. A high AL was defined as AL > median. Logistic regression analyses examined the association between AL and BRCL, adjusting for sociodemographic, clinical, and treatment factors.

**Results:**

Among 3,609 patients, 18.86% (n = 681) developed lymphedema. A higher proportion of patients with lymphedema were Black (11.89% vs. 7.38%, p < 0.0001), Medicaid insured (12.19% vs. 6.97%, p < 0.0001), had stage 3 disease (7.05% vs. 1.57%, p < 0.0001), and had a high AL (53.63% vs. 46.90%, p = 0.0018). In adjusted analysis, high AL was associated with higher odds of developing lymphedema than low AL (OR 1.281 95% CI 1.06–1.55). Moreover, a 1-unit increase in AL was associated with 10% higher odds of lymphedema (OR 1.10, 95% CI 1.04–1.16). There was no statistically significant association between AL and severity of lymphedema (OR 1.02, 95% CI 0.82–1.23).

**Conclusion:**

In this retrospective cohort of breast cancer survivors, high AL at diagnosis was associated with higher odds of developing lymphedema. Future research should elucidate the pathways by which AL influences lymphedema.

**Supplementary information:**

The online version contains supplementary material available at 10.1007/s00520-025-09362-4.

## Introduction

Breast cancer-related lymphedema (BCRL) is a common debilitating complication following breast cancer treatment, with incidence rates ranging between 5–40% depending on risk factors such as obesity, infections, receipt of radiation therapy, chemotherapy, and extent of axillary lymph node dissection [1–3]. Recent studies have highlighted the influence of socioenvironmental stressors, defined as the social and physical aspects of an individual’s living and working conditions, on the development and severity of BCRL[4, 5]. For example, rurality, low income, and poor social support have been associated with the development of BCRL [4, 5]. Additionally, comorbidities such as obesity, which have previously been linked to adverse socioenvironmental stressors, have also been associated with a higher risk of developing BCRL [6, 7]. Nonetheless, despite growing evidence suggesting an association between socioenvironmental stressors and lymphedema, there is a paucity of research examining the biological correlates of these stressors, such as allostatic load, in the context of BCRL [8].

**Fig.1:**
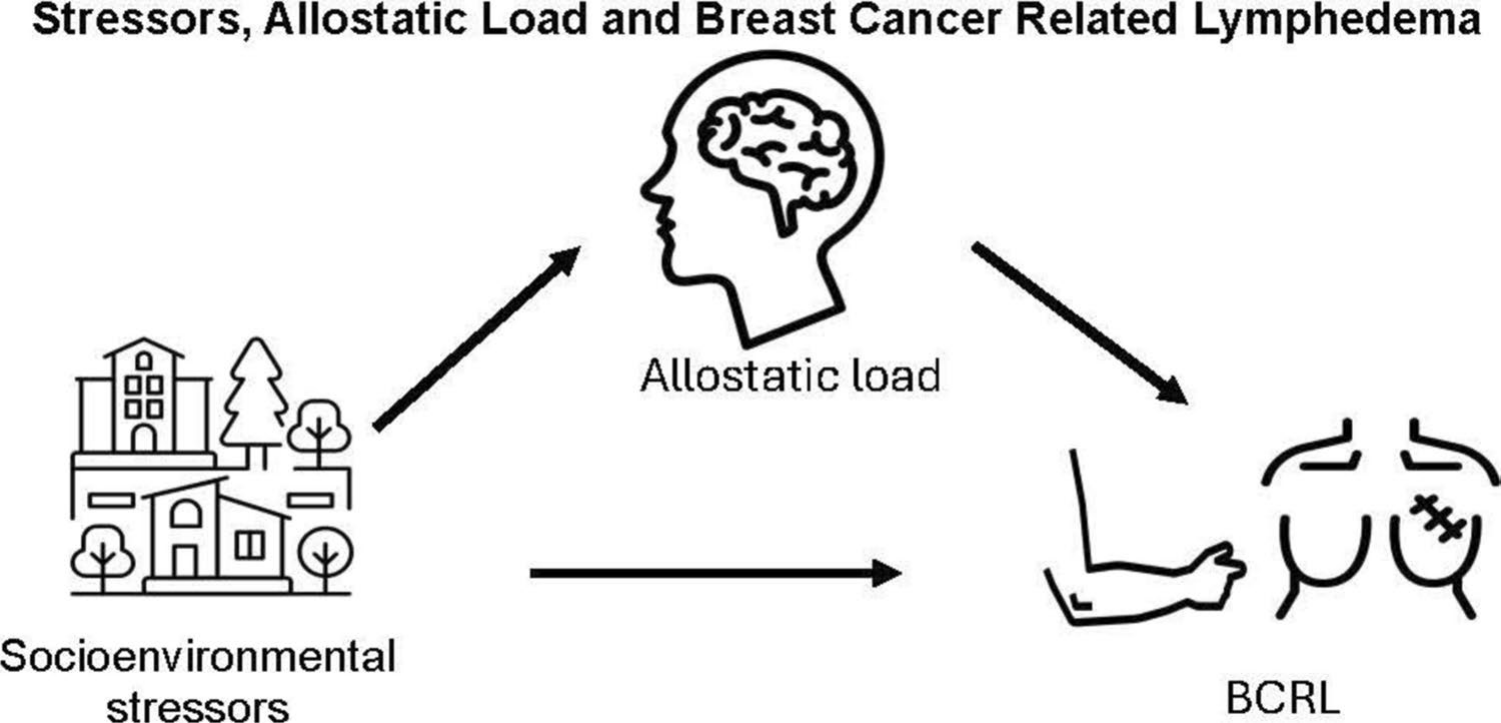
Conceptual Framework of the Association between Socioenvironmental Stressors, Allostatic Load and Breast Cancer Related Lymphedema

Allostatic load (AL) is a multisystem construct incorporating biomarkers from various physiological domains, including the metabolic, neuroendocrine, immune, and cardiovascular systems, to measure physiological dysregulation secondary to chronic activation of the hypothalamic–pituitary–adrenal (HPA) axis and the sympathetic-adrenal-medullary (SAM) pathway [9]. AL has been linked to various adverse health outcomes, including cancer [10–13]. Previous studies have demonstrated that high AL, an indicator of greater physiologic dysregulation, is associated with more advanced disease severity and increased all-cause mortality in patients with breast cancer and other solid tumors [14–16]. Moreover, elevated AL has been associated with socioenvironmental factors such as race, socioeconomic status, and neighborhood characteristics [14, 16]. Our recent examination of AL and 30-day postoperative complications in patients with breast cancer showed an association between high AL and higher rates of developing postoperative complications [17]. Furthermore, mediation analysis suggested that AL may influence the development of postoperative complications indirectly through comorbidities and directly through an independent pathway [17]. Taken together, these studies suggest AL might be a plausible biological intermediary between socioenvironmental stressors and the development of chronic post-treatment complications such as BCRL **(**Fig. 1).

The objective of this study was to understand the association between AL at diagnosis and lymphedema development in a cohort of breast cancer survivors who received surgical treatment. We hypothesize that patients with high allostatic load scores at diagnosis will have higher odds of developing lymphedema than those with low allostatic load scores.

## Methods

### Data source

**Fig. 2:**
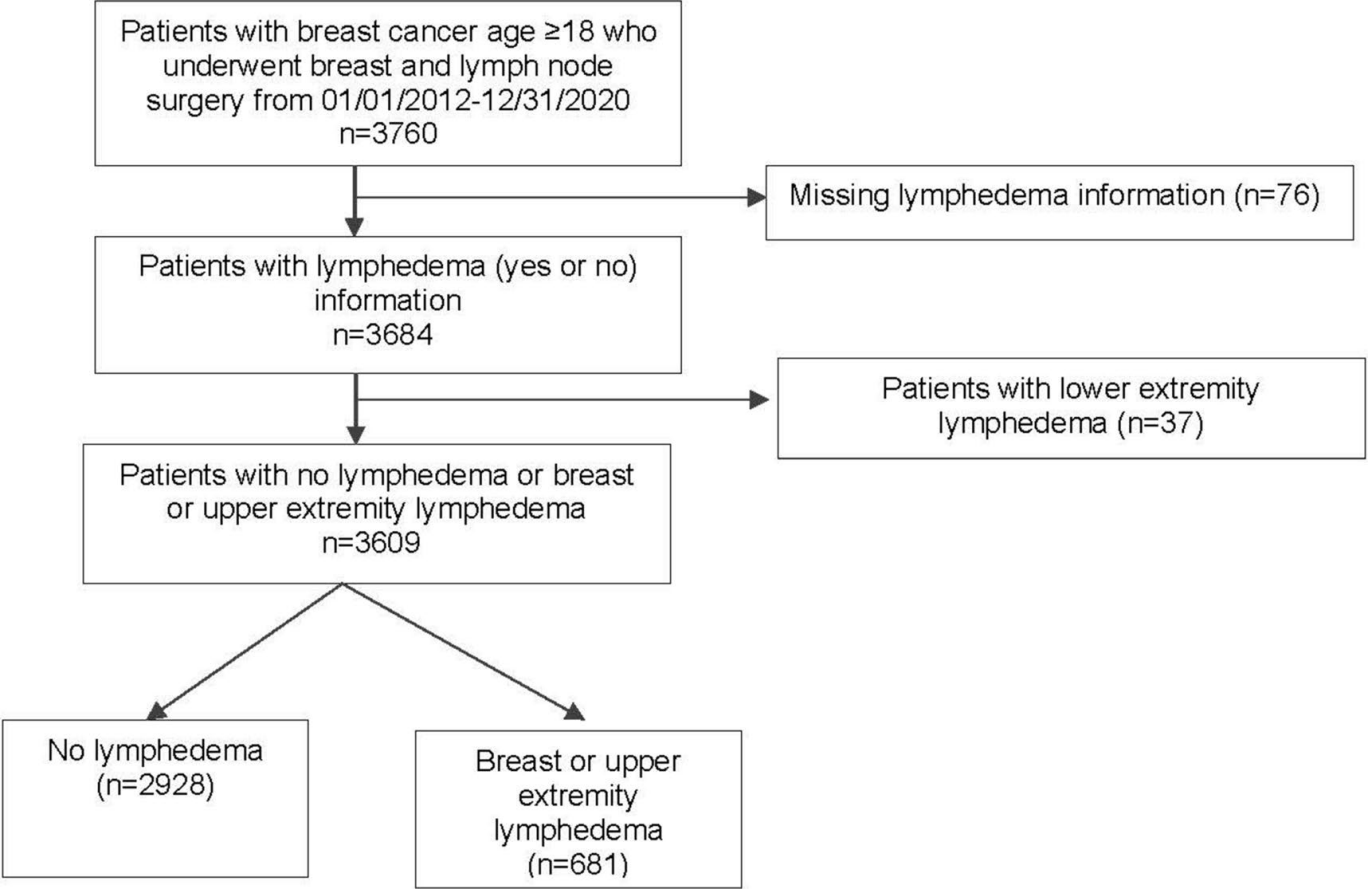
Study Schema

Women diagnosed with stage I-III breast cancer from January 1st, 2012, through December 31st, 2020, who underwent operative management were identified in The Ohio State University Cancer registry and The Ohio State University Comprehensive Cancer Center electronic medical records (IHIS). Patients diagnosed with ductal carcinoma in situ (stage 0), metastatic disease (stage IV), recurrent breast cancer, unknown breast cancer subtype, and individuals who did not receive surgical management, had missing lymphedema information, or had lower extremity lymphedema were excluded. (Fig. 2).

### Study measures

#### Sociodemographic characteristics

Sociodemographic variables included age, race (White, Black, or Other), ethnicity (Hispanic or non-Hispanic), marital status (single, married/living as married or widowed/separated/divorced), insurance (Private, Medicaid, Medicare, Other), smoking history (yes or no) and alcohol use (yes or no). All racial and ethnic categories are self-reported. Patients who self-identified as Asian, American Indian, Alaskan Native, Other Pacific Islander, Native Hawaiian, or multiracial were collapsed into the “Other” category due to small sample sizes. In this study, race is a sociopolitical construct, not a measure of genetic ancestry [18]. The Charlson Comorbidity Index (CCI) was categorized into 0, 1–3, or 4 + and was modified to exclude cancer as a contributor to comorbidity [19].

#### Clinical and treatment characteristics

Patient stage (clinical and pathological), hormone receptor status (estrogen and progesterone receptors [HR]), and human epidermal growth factor receptor 2 (HER2) status were obtained from the electronic medical record. Molecular subtypes were categorized into HR positive/HER2 negative (HR + /HER2-), HR + /HER2 + , HR-/HER2 + or HR-/HER2-. Breast (mastectomy, lumpectomy, or both) and axillary (sentinel lymph node biopsy (SLNB) only, axillary lymph node dissection (ALND) only, or both SLNB and ALND) surgeries were included. Chemotherapy and radiation therapy were dichotomized as yes or no.

#### Allostatic load

There is currently no consensus on standard biomarkers for calculating allostatic load[20]. This study uses biomarkers across four physiologic systems commonly reported in the literature: cardiovascular (heart rate (HR), systolic (SBP) and diastolic (DBP) blood pressures), metabolic (body mass index (BMI), alkaline phosphatase (ALP), glucose, albumin), renal (creatinine and blood urea nitrogen (BUN)), and immunologic (white blood cell count (WBC))[20]. Biomarkers were collected up to 12 months before or 6 months following biopsy-proven cancer diagnosis in the electronic medical record. Distributions of these biomarkers were divided into quartiles, and values in the worst quartile of the cohort were given a point [21]. Specifically, biomarkers ≥ 75th percentile for HR, SBP, DBP, ALP, glucose, creatinine, BUN, and WBC were each given a point. In contrast, albumin ≤ 25th percentile was assigned a point. Patients with a BMI of < 18.5 or ≥ 30 were also given a point. Points were summed and then dichotomized into low vs. high based on the median sum score (AL 2.0) and categorized into quartiles (Q1 with sum 0–1, Q2 with sum 2, Q3 with sum 3–4, Q4 with sum 5 +). Higher scores were indicative of worse physiological disturbance.

#### Study outcome––lymphedema

The primary outcome was the development of breast or ipsilateral upper extremity lymphedema (yes/no). Patients with a history of breast cancer diagnosed between 01/01/2012 and 12/31/2020 who developed lymphedema following their initial treatment (surgery or chemotherapy) were identified through the electronic medical record (EMR). Specifically, a data analyst queried patient notes for the term “lymphedema staging,” and physical therapy notes were subsequently reviewed to confirm the diagnosis and severity of lymphedema. Individuals with either breast-only or upper-extremity lymphedema were coded as ‘yes’ for lymphedema. Patients with lower extremity lymphedema were excluded from the study. The highest grade was selected for patients with multiple grades. Grades were dichotomized as 0/1 or 2/3.

### Statistical analysis

Multiple imputations by chained equations were used to account for missing values [22]. Ten imputed datasets were created using logistic regression-based imputation models for binary and ordinal variables, discriminant function for non-ordered variables, and regression-based projected mean matching for continuous variables. Imputation-corrected parameters and standard errors (SEs) were combined using Rubin’s rule[23].

Sociodemographic, clinical, and treatment variables were summarized using descriptive statistics, with medians and interquartile ranges (IQRs) for continuous variables and frequencies and percentages for categorical variables. Differences between groups based on lymphedema development were calculated using Wilcoxon rank-sum, chi-square, or Fisher’s exact test.

Crude and adjusted logistic regression analyses were performed to test the association between AL and the development of lymphedema. In addition, among patients who developed lymphedema, crude and adjusted models assessed lymphedema grade (2/3 vs. 0/1) as the outcome with AL as the exposure. To increase the comparability of our results, we included AL as a continuous, binary, and quartile variable in all fitted regression models.

Secondary analysis examined the relationship between lymphedema and each of the 10 AL biomarkers using established clinical cut-offs [16]. To examine the value of the composite AL as an independent predictor of lymphedema, univariate and multivariable binary logistic regressions modeled each AL biomarker and the composite AL score (as a 1-unit increase).

All analyses were performed using the SAS software (version 9.4; SAS Institute, Cary, NC, USA). A two-sided p-value of less than 0.05 was considered statistically significant. The authors used ChatGPT and Grammarly to improve the language and readability during the preparation of this manuscript.

## Results

### Description of study cohort

3,609 patients met the study criteria, of whom 18.86% (n = 681) developed lymphedema. Patients who developed BCRL were younger than those who did not develop lymphedema (lymphedema 55.2 years (interquartile range (IQR) 47.1 years-64.0y ears, no lymphedema 59.1 years (IQR 49.7 years −67.1 years). Compared with patients without lymphedema, a higher proportion of patients who developed lymphedema were racialized as Black (lymphedema 11.89% vs. no lymphedema 7.38%, p < 0.0001) and had Medicaid insurance (12.19% vs. 6.97%, p < 0.0001) **(**Table 1**)**. Additionally, patients who developed lymphedema had more aggressive breast cancer molecular subtypes (p = 0.0033) and higher disease stages (p < 0.0001) than those without lymphedema. A higher percentage of patients with lymphedema received chemotherapy (lymphedema 63.14% vs. no lymphedema 37.77%, p < 0.0001), radiation therapy (lymphedema 66.62% vs. no lymphedema 57.82%, p < 0.01) and underwent ALND (lymphedema 67.11% vs. 5.82%, p < 0.0001). There was no significant difference in the CCI based on lymphedema development (p = 0.515).

**Table 1:** Sociodemographic, clinical and treatment characteristics based on lymphedema development

Patient Characteristics, n (%)	Total Sample (N = 3609)	Lymphedema (n = 681)	No Lymphedema (n = 2928)	P-value
Age at diagnosis (in years) Median (Q_1_, Q_3_)	58.5 (49.0, 66.5)	55.2 (47.1, 64.0)	59.1 (49.7, 67.1)	< 0.0001
Race White Black Other	3166 (87.73%) 297 (8.23%) 146 (4.05%)	563 (82.76%) 81 (11.89%) 37 (5.43%)	2603 (88.90%) 216 (7.38%) 109 (3.72%)	< 0.0001
Ethnicity Hispanic Non-Hispanic	40 (1.11%) 3569 (98.89%)	12 (1.76%) 669 (98.19%)	28 (0.96%) 2990 (99.04%)	0.0704
Marital Status Single Married/living as married Widowed, separated or divorced	499 (13.83%) 2328 (64.51%) 782 (21.67%)	107 (15.71%) 433 (63.58%) 141 (20.70%)	392 (13.39%) 1895 (64.72%) 641 (21.89%)	0.2686
Insurance Private Medicaid Medicare Other	2159 (59.82%) 287 (7.95%) 1114 (30.87%) 49 (1.36%)	422 (61.97%) 83 (12.19%) 167 (24.52%) 9 (1.32%)	1737 (59.32%) 204 (6.97%) 947 (32.34%) 40 (1.37%)	< 0.0001
Smoking History Never Current or Former	2254 (62.44%) 1355 (37.56%)	441 (64.79%) 240 (35.21%)	1812 (61.90%) 1116 (38.10%)	0.2468
Alcohol Use Never Current or former	1686 (46.72%) 1923 (53.28%)	355 (52.09%) 326 (47.91%)	1331 (45.47%) 1597 (54.53%)	0.0019
Charlson Comorbidity index 0 1–3 ≥ 4	2879 (79.77%) 655 (18.15%) 75 (2.08%)	541 (79.44%) 122 (17.91%) 18 (2.64%)	2338 (79.85%) 533 (18.20%) 57 (1.95%)	0.5152
Estrogen receptor status Negative Positive	662 (18.34%) 2947 (81.66%)	155 (22.76%) 526 (77.24%)	507 (17.32%) 2421 (82.68%)	0.0009
Progesterone receptor Negative Positive	1013 (28.07%) 2596 (71.93%)	222 (32.60%) 459 (67.40%)	791 (27.02%) 2137 (72.98%)	0.0035
Subtype HR + /HER2- HR + /HER2 + HR-/HER2- HR-/HER2 +	2303 (63.81%) 645 (17.87%) 495 (13.72%) 166 (4.60%)	399 (58.59%) 127 (18.65%) 120 (17.62%) 35 (5.14%)	1904 (65.03%) 518 (17.69%) 375 (12.81%) 131 (4.47%)	0.0033
Cancer Stage 1 2 3	2497 (69.19%) 1018 (28.21%) 94 (2.60%)	357 (52.42%) 276 (40.53%) 48 (7.05%)	2140 (73.09%) 742 (25.34%) 46 (1.57%)	< 0.0001
Chemotherapy Yes No	1536 (42.56%) 2073 (57.44%)	430 (63.14%) 251 (36.86%)	1106 (37.77%) 1822 (62.23%)	< 0.0001
Radiation Therapy Yes No	2150 (59.57%) 1459 (40.43%)	457 (67.11%) 224 (32.89%)	1693 (57.82%) 1235 (42.18%)	< 0.0001
Breast surgery type Mastectomy Lumpectomy Both	1467 (40.65%) 2032 (56.30%) 110 (3.05%)	327 (48.02%) 319 (46.84%) 35 (5.14%)	1140 (38.93%) 1713 (58.50%) 75 (2.56%)	< 0.0001
Lymph node surgery Sentinel lymph node biopsy only Axillary lymph node dissection only Both SLNB + ALND	1411 (39.10%) 226 (6.26%) 1972 (54.64%)	159 (23.35%) 132 (19.38%) 390 (57.27%)	1252 (42.76%) 94 (3.21%) 1582 (54.03%)	< 0.0001
Allostatic load Median (IQR)	1.92 (0.80–3.15)	2.18 (1.02–3.49)	1.87 (0.76–3.05)	0.0001
Allostatic load (binary) Low (≤ 2) High (> 2)	1871 (51.84%) 1738 (48.16%)	316 (46.42%) 365 (53.58%)	1555 (53.10%) 1373 (46.90%)	0.0025
Allostatic load (quartiles) Q1 (0–1) Q2 (2) Q3 (3–4) Q4 (5 +)	1035 (28.69%) 836 (23.16%) 1266 (35.09%) 472 (13.07%)	168 (24.65%) 148 (21.76%) 256 (37.61%) 103 (15.98%)	867 (29.62%) 688 (23.48%) 1010 (34.50%) 363 (12.39%)	0.0076

### Allostatic load and lymphedema

As a binary variable (low vs. high AL), a higher proportion of patients who developed lymphedema had higher AL (53.58%) than those without lymphedema (46.90%, p < 0.001). Patients with high AL had 28% higher odds of developing lymphedema (OR = 1.28, 95% CI: 1.06–1.55) than those with low AL (Table 2). Similarly, patients in the highest AL quartile (Q4 OR = 1.51, 95% CI: 1.09–2.08) had 51% higher odds of developing lymphedema than those in the lowest AL quartile (Q1). A one-unit increase in AL was associated with 10% higher odds of lymphedema (OR = 1.10, 95% CI: 1.04–1.16). There was no significant association between the lymphedema grade and AL (Table 3). After controlling for individual AL biomarkers, sociodemographic characteristics, and treatment receipt, composite AL and BMI were the only biomarker variables significantly associated with lymphedema (Supplementary Table 1).

**Table 2:** Association between AL and development of lymphedema (n = 3,609)*

	Crude OR (95% CI)	Adjusted OR (95% CI)
AL (continuous) 1-unit increase	1.10 (1.05–1.16)	1.10 (1.04–1.16)
AL (in quartiles) Q1 (0–1) Q2 (2) Q3 (3–4) Q4 (5 +)	Ref 1.11 (0.87–1.43) 1.31 (1.05–1.64) 1.55 (1.16–2.06)	Ref 1.09 (0.84–1.43) 1.28 (1.00–1.64) 1.51 (1.09–2.08)
AL (binary) Low (≤ 2) High (> 2)	Ref 1.31 (1.10–1.56)	Ref 1.28 (1.06–1.55)

^*^Adjusted for age, race, chemotherapy, radiation therapy, breast surgery type, and lymph node surgery type

**Table 3:** Association between AL and Lymphedema Grade (n = 226)*

	Crude OR (95% CI)	Adjusted OR (95% CI)
AL (continuous) 1-unit increase	1.07 (0.91–1.26)	1.02 (0.85–1.23)
AL (in quartiles) Q1 (0–1) Q2 (2) Q3 (3–4) Q4 (5 +)	Ref 0.91 (0.40–2.10) 1.64 (0.80–3.34) 1.20 (0.51–2.79)	Ref 0.86 (0.35–2.12) 1.60 (0.73–3.50) 0.92 (0.36–2.35)
AL (binary) Low (≤ 2) High (> 2)	Ref 1.56 (0.91–2.68)	Ref 1.46 (0.80–2.67)

^*^Adjusted for age, race, chemotherapy, radiation therapy, breast surgery type and lymph node surgery

Of note, consistent with existing literature, patients with high AL in this study cohort were more likely to be older, racialized as Black, Medicaid insured, unpartnered, have more aggressive molecular subtypes, higher stages of disease, and a higher CCI than those with low AL **(**Supplementary Table 2**).**

## Discussion

In this large retrospective analysis of patients who received surgical management for breast cancer, elevated allostatic load was associated with a higher probability of developing BCRL. Moreover, in models controlling for individual AL biomarkers, high AL remained significantly associated with the development of BCRL. These results suggest that AL may contribute to the development of BCRL independent of its individual biomarker components.

The pathophysiology of BCRL suggests that the extent of axillary surgery, radiation therapy, and receipt of taxane chemotherapy cause mechanical disruptions or pathologic changes (e.g., fibrosis) to the lymphatic system, resulting in lymphedema [3, 24]. A plausible mechanistic pathway of how AL influences lymphedema development is concomitantly through its individual biomarkers and as a common factor[25, 26]. For example, biomarkers frequently used to calculate AL, such as albumin, blood glucose, inflammatory biomarkers (e.g., interleukin 6) and BMI have all been implicated in wound healing or BCRL [27–31]. Similarly, our supplementary analysis showed an association between 1) AL and BCRL, and 2) BMI and BRCL after controlling for the individual biomarkers used to calculate AL. Additionally, in our prior examination of AL and acute post-operative complications (e.g., hematoma) in patients with breast cancer, AL was associated with the development of postoperative complications directly and indirectly through a shared pathway with comorbidities [17, 32]. This is particularly relevant, as Konishi et al.’s study on factors associated with arm lymphedema post-surgery found that postoperative bleeding increases the likelihood of arm lymphedema [33]. Collectively, these studies suggest AL may work through a bifactor model where AL contributes to treatment-related complications independent of its constituent components [25, 26].

The weathering hypothesis and ecosocial theory may provide conceptual frameworks for the internalization of socioenvironmental stressors through mechanisms such as AL. Both conceptual frameworks postulate that chronic exposure to adverse socioenvironmental stressors results in physiologic and molecular changes with implications for disease distribution within populations [34, 35]. For patients with breast cancer, studies indicate that exposure to adverse socioenvironmental factors, such as low educational attainment, lack of medical insurance, and low income, are associated with BCRL development [36]. In this study, a higher percentage of Black and Medicaid-insured patients had lymphedema compared to other racial categories or insurance types. Also consistent with existing literature, patients encountering adverse socioenvironmental conditions, such as Black women, those with Medicaid insurance, and unpartnered individuals, were more likely to have high AL compared to White women, those with private insurance, and those who were partnered [14, 16, 37, 38]. Overall, this study's findings and existing literature highlight relationships between environmental exposures, their impact on physiology, and potential implications for treatment outcomes.

The percentage of patients developing lymphedema in this study is consistent with prior studies examining lymphedema in patients with breast cancer. In their systematic review and meta-analysis of lymphedema after breast cancer, Disipio et al. estimated an overall BCRL incidence of 16.6% and an incidence range between 8.4% and 24.1% for prospective studies[1]. Similarly, in this study, approximately 19% of patients developed BCRL. Patient (e.g., Black race) characteristics and treatment-related factors (e.g., undergoing axillary lymph node dissection) more common in patients with BCRL were also consistent with existing literature[39, 40].

The strengths of this study include a large, diverse patient cohort and the use of a robust measure of AL that has been used in multiple studies [14, 15, 17, 41]. Moreover, the AL biomarkers used in this study are routinely collected in the clinical care of patients with cancer, allowing others to easily replicate this study in other cancer populations. Secondary analysis confirmed AL was associated with the outcome of interest even after controlling for individual AL biomarkers and sociodemographic, clinical, and treatment factors, enhancing our findings' internal validity.

However, the study also has some limitations. As a single-institution retrospective study, the generalizability of our findings may be limited. Additionally, the retrospective nature introduces potential selection biases. The use of the electronic medical record introduces additional limitations, as it may not capture care provided outside of OSUCCC and is susceptible to exposure misclassification [42]. Future prospective, multi-center studies are needed to validate our findings and explore the mechanisms underlying the association between AL and BCRL.

## Conclusion

This study provides novel evidence of the association between AL and BCRL development. Moreover, it highlights the importance of considering the impact of chronic exposure to socioenvironmental stressors, potentially manifesting in measures such as AL, in the context of lymphedema. Future research should focus on elucidating the specific pathways through which AL influences lymphedema development and evaluating the effectiveness of AL-targeted interventions in reducing BCRL risk and improving outcomes for breast cancer survivors.

## Supplementary information

Below is the link to the electronic supplementary material.

ESM 1 (DOCX 24.7 KB)

## Data Availability

The data from this study includes Health Insurance Portability and Accountability Act data and cannot be publicly shared.
