# Supplementary material for: The association between allostatic load and lymphedema in breast cancer survivors
Source: Support Care Cancer. 2025 Mar 21;33(4):311. doi: 10.1007/s00520-025-09362-4 (PMC11928421; doi:10.1007/s00520-025-09362-4)
Supplement: Supplementary file 1 — (DOCX 24.7 KB) [file 520_2025_9362_MOESM1_ESM.docx]

**Supplementary Tables**

| **Supplementary Table 1. Association between Composite AL, Individual AL Biomarkers and Lymphedema** | | |
| --- | --- | --- |
| Biomarkers | Cutoff Units | Adjusted† |
|  |  | OR (95% CI) |
| Allostatic Load Score | 1-unit increase | 1.08 (1.01-1.15) |
| Alkaline Phosphatase | >126 U/L | 0.73 (0.36-1.50) |
| Albumin | <3.5 g/dL | 1.12 (0.49-2.57) |
| Serum Creatinine | >1.3 mg/dL | 1.07 (0.58-1.96) |
| Heart Rate | >100 | 1.02 (0.70-1.51) |
| White Blood Cell Count | >11.19 K/uL | 0.95 (0.65-1.38) |
| Body Mass Index | <18.5 or >25 Kg/m² | 1.51 (1.19-1.92) |
| Blood Pressure Diastolic | ≥90 mmHg | 0.91 (0.71-1.19) |
| Blood Pressure Systolic | ≥130 mmHg | 1.00 (0.80-1.24) |
| Blood Urea Nitrogen | >25 mg/dL | 0.80 (0.43-1.49) |
| Glucose | ≥100 mg/dL | 1.05 (0.86-1.28) |
| †Adjusted for total allostatic load score (range 0 to 10), alkaline phosphatase, albumin, creatinine serum, heart rate, white blood cell count, body mass index (BMI), blood pressure diastolic, blood pressure systolic, blood urea nitrogen and glucose as well as age, race, chemotherapy, radiation therapy, breast surgery type and lymph node surgery. | | |
| OR=Odds Ratio; CI=Confidence Interval | | |

| **Supplementary Table 2: Sociodemographic, Clinical and Treatment Characteristics based on Allostatic Load Scores** | | | | |
| --- | --- | --- | --- | --- |
| Patient Characteristics | **Total Sample**  **(N=3609)** | **High AL**  **(n=1738)** | **Low AL**  **(n=1871)** | **P-value** |
| Age at biopsy (in years)  Median (Q_1_, Q_3_) | 58.5 (49.0, 66.5) | 60.3 (51.2, 67.9) | 56.7 (47.5, 65.0) | <0.0001 |
| Race  White  Black  Other | 3166 (87.73%)  297 (8.23%)  146 (4.05%) | 1510 (86.89%)  180 (10.34%)  48 (2.77%) | 1656 (88.50%)  117 (6.27%)  98 (5.23%) | <0.0001 |
| Ethnicity  Hispanic  Non-Hispanic | 40 (1.11%)  3569 (98.89%) | 18 (1.03%)  1720 (98.97%) | 22 (1.18%)  1849 (98.82%) | 0.6706 |
| Marital Status  Single  Married/living as married  Widowed, separated or divorced | 499 (13.83%)  2328 (64.51%)  782 (21.67%) | 244 (14.01%)  1047 (60.24%)  448 (25.75%) | 256 (13.66%)  1281 (68.47%)  335 (17.88%) | <0.0001 |
| Insurance  Private  Medicaid  Medicare  Other | 2159 (59.82%)  287 (7.95%)  1114 (30.87%)  49 (1.36%) | 925 (53.23%)  146 (8.42%)  645 (37.12%)  21 (1.23%) | 1234 (65.95%)  141 (7.51%)  469 (25.06%)  28 (1.48%) | <0.0001 |
| Smoking History  Never  Current or Former | 2254 (62.44%)  1355 (37.56%) | 1057 (60.80%)  681 (39.20%) | 1197 (63.97%)  674 (36.03%) | 0.1113 |
| Alcohol Use  Never  Current or former | 1686 (46.72%)  1923 (53.28%) | 922 (53.03%)  816 (46.97%) | 764 (40.85%)  1107 (59.15%) | <0.0001 |
| Charlson Comorbidity index  0  1-3  ≥4 | 2879 (79.77%)  655 (18.15%)  75 (2.08%) | 1271 (73.10%)  411 (23.67%)  56 (3.23%) | 1609 (85.97%)  244 (13.02%)  19 (1.01%) | <0.0001 |
| Subtype  HR+/HER2-  HR+/HER2+  HR-/HER2-  HR-/HER2+ | 2303 (63.81%)  645 (17.87%)  495 (13.72%)  166 (4.60%) | 1074 (61.81%)  319 (18.34%)  269 (15.45%)  77 (4.40%) | 1229 (65.67%)  326 (17.44%)  227 (12.11%)  90 (4.78%) | 0.0263 |
| Cancer Stage  1  2  3 | 2497 (69.19%)  1018 (28.21%)  94 (2.60%) | 1154 (66.37%)  534 (30.74%)  50 (2.89%) | 1344 (71.81%)  484 (25.85%)  44 (2.34%) | 0.0038 |
| Chemotherapy  Yes  No | 1536 (42.56%)  2073 (57.44%) | 783 (45.06%)  955 (54.94%) | 753 (40.24%)  1118 (59.76%) | 0.0051 |
| Radiation Therapy  Yes  No | 2150 (59.57%)  1459 (40.43%) | 1063 (61.17%)  675 (38.83%) | 1087 (58.09%)  784 (41.91%) | 0.0709 |
| Breast surgery type  Mastectomy  Lumpectomy  Both | 1467 (40.65%)  2032 (56.30%)  110 (3.05%) | 666 (38.31%)  1016 (58.43%)  57 (3.25%) | 801 (42.82%)  1016 (54.32%)  54 (2.86%) | 0.0290 |
| Lymph node surgery  Sentinel lymph node biopsy only  Axillary lymph node dissection only  Both SLNB +ALND | 1411 (39.10%)  226 (6.26%)  1972 (54.64%) | 671 (38.61%)  122 (7.00%)  945 (54.40%) | 740 (39.55%)  104 (5.58%)  1027 (54.87%) | 0.2436 |
